# Intraoperative end-tidal carbon dioxide levels are not associated with recurrence-free survival after elective pancreatic cancer surgery: a retrospective cohort study

**DOI:** 10.3389/fmed.2024.1442283

**Published:** 2024-09-11

**Authors:** Sarah Dehne, Lina Kirschner, Rosa Klotz, Samuel Kilian, Christoph W. Michalski, Thilo Hackert, Markus W. Büchler, Markus A. Weigand, Jan Larmann

**Affiliations:** ^1^Heidelberg University, Medical Faculty Heidelberg, Department of Anesthesiology, Heidelberg, Germany; ^2^Heidelberg University, Medical Faculty Heidelberg, Department of General, Visceral, and Transplantation Surgery, Heidelberg, Germany; ^3^Heidelberg University, Medical Faculty Heidelberg, Institute of Medical Biometry, Heidelberg, Germany

**Keywords:** carbon dioxide, pancreatic cancer surgery, oncological outcome, recurrence-free survival, perioperative complications

## Abstract

**Background:**

Intraoperative end-tidal carbon dioxide concentrations (EtCO_2_) values are associated with recurrence-free survival after colorectal cancer surgery. However, it is unknown if similar effects can be observed after other surgical procedures. There is now evidence available for target EtCO_2_ and its relation to surgical outcomes following pancreatic cancer surgery.

**Methods:**

In this single-center, retrospective cohort study, we analyzed 652 patients undergoing elective resection of pancreatic cancer at Heidelberg University Hospital between 2009 and 2016. The entire patient cohort was sorted in ascending order based on mean intraoperative EtCO_2_ values and then divided into two groups: the high-EtCO_2_ group and the low-EtCO2 group. The pre-specified primary endpoint was the assessment of recurrence-free survival up to the last known follow-up. Cardiovascular events, surgical site infections, sepsis, and reoperations during the hospital stay, as well as overall survival were pre-specified secondary outcomes.

**Results:**

Mean EtCO_2_ was 33.8 mmHg ±1.1 in the low-EtCO_2_ group vs. 36.8 mmHg ±1.9 in the high-EtCO_2_ group. Median follow-up was 2.6 (Q1:1.4; Q3:4.4) years. Recurrence-free survival did not differ among the high and low-EtCO_2_ groups [HR = 1.043 (95% CI: 0.875–1.243), log rank test: *p* = 0.909]. Factors affecting the primary endpoint were studied via Cox analysis, which indicated no correlation between mean EtCO_2_ levels and recurrence-free survival [Coefficient −0.004, HR = 0.996 (95% CI:0.95–1.04); *p* = 0.871]. We did not identify any differences in the secondary endpoints, either.

**Conclusions:**

During elective pancreatic cancer surgery, anesthesiologists should set EtCO_2_ targets for reasons other than oncological outcome until conclusive evidence from prospective, multicenter randomized controlled trials is available.

## 1 Introduction

During surgery, the measurement of end-tidal carbon dioxide concentration (EtCO_2_) serves as a non-invasive method to estimate arterial carbon dioxide pressure (PaCO_2_) (1), reflecting the amount of carbon dioxide (CO_2_) dissolved in arterial blood. CO_2_ can impact cellular mechanism such as cell movement, apoptosis, and cell growth (2–5). *In vitro*, elevated CO_2_ levels may enhance the invasive capabilities of various cancer cells (6–9). In colon cancer cells, exposure to CO_2_ exhibits in heightened proliferation, adhesion disorder and elevated levels of growth factor expression (7, 10). Effects of CO_2_ exposure on tumor biology have also been demonstrated in pancreatic ductal adenocarcinoma cells. Exposure to hypercapnia can result in increased cell colony formation, elevated cell division process, and higher radio- and chemotherapy resistance (11, 12).

During surgical procedures, EtCO_2_ is influenced by mechanical ventilation and typically ranges from 30 to 45 mmHg (13–15). Currently, there are no recommendations for precise intraoperative target EtCO_2_ levels derived from outcome studies (2). Intraoperative EtCO_2_ levels are influenced by several factors, such as the presence of existing pulmonary conditions, the kind of surgery, and the anesthesiologist's preferences (2).

In our retrospective cohort study involving 528 patients undergoing colorectal cancer surgery, lower EtCO_2_ values were associated with enhanced oncological outcome (16). Since *in vitro* experiments suggest that CO_2_ may potentially affect the tumor biology of pancreatic cancer cells and because the impact of different intraoperative EtCO_2_ values on oncological outcome in patients undergoing pancreatic cancer surgery is unknown, we performed this retrospective cohort study to examine the association between intraoperative EtCO_2_ levels and recurrence-free survival.

## 2 Methods

### 2.1 Study design and cohort

A retrospective cohort study was conducted involving patients who received general anesthesia for elective pancreatic cancer surgery at the Department of General, Visceral, and Transplant Surgery, Heidelberg University Hospital, Heidelberg, Germany.

Our study protocol (S-723/2021) received approval by the local Ethics Committee of the Medical Faculty of Ruprecht-Karls-University, Heidelberg, Germany, on 29 March, 2022. The principles described in the Declaration of Helsinki and the STROBE guidelines for observational studies have been followed in preparing this report (17).

We evaluated the association between intraoperative EtCO_2_ levels and recurrence-free survival following pancreatic cancer surgery. Patients who underwent pancreatic cancer surgery between 2009 and 2016 were analyzed. Only patients ≥18 years of age with a minimum of 180 days of follow-up data were eligible for inclusion. Patients with distant metastases at the time of surgery were not included in this study. Furthermore, the histopathological examination following elective pancreatic tumor surgery had to show either an R1 result (microscopic residual tumor) or an R0 result (no residual tumor) for inclusion. Exclusion criteria included identification of peritoneal carcinomatosis during surgery or cases where histological analysis could not verify the presence of cancer tissue, such as those following neoadjuvant chemotherapy or radiotherapy.

### 2.2 Data collection

Data were accessed from patients' medical records and the prospectively-maintained electronic databases of the Department of Surgery at Heidelberg University Hospital. Data incorporated demographic information, American Society of Anesthesiologists physical status classification (ASA), body mass index (BMI), pre-existing conditions, neoadjuvant and adjuvant therapy, duration of surgery, intraoperative opioid usage and transfusions, performance of intraoperative radiation therapy (IORT), duration of hospital and intensive care unit stay, and outcome parameters. Resection margin status, tumor grade, and TNM (tumor, node, metastasis) classification were obtained from pathology findings. The mean EtCO_2_ was determined using EtCO_2_ values documented in the anesthesia records every quarter hour from intubation to extubation throughout the entire surgery. The EtCO_2_ was measured in real-time using infrared spectroscopy in the “main stream” method. The documented EtCO_2_ values correspond to the plateau values of the EtCO_2_ curves displayed by the ventilator.

### 2.3 Outcome analysis

The primary outcome measure was recurrence-free survival in the period from index surgery until the last known follow-up, with a median follow-up duration of 2.6 years (Q1:1.4; Q3:4.4). Recurrence-free survival was defined as the time from index surgery to the first documented event of local cancer recurrence, newly diagnosed metastases, or death. During the follow-up examinations, abdominal ultrasounds, computer tomographies, physical examinations and blood samples were carried out at regular intervals or when new suspicious symptoms occurred. If there were no documented instances of cancer recurrence, new metastases, or death, we recorded the date of the last follow-up or doctor-patient contact with negative findings. Surgical site infections (SSI), sepsis, reoperation due to surgical complications, and cardiovascular events (transitory ischemic attack, and cerebral- or myocardial infarction) during the hospital stay and overall survival were secondary outcomes.

### 2.4 Statistical analysis

The entire patient cohort was sorted in ascending order based on mean intraoperative EtCO_2_ values and then divided into two groups: the high-EtCO_2_ group and the low-EtCO_2_ group, each consisting of 326 patients. Descriptive analyses involved calculating the mean, standard deviation (SD), median, and first and third quartiles for continuous variables, and absolute and relative proportions for categorical variables. The chi-square test was employed to compare the distribution of categorical variables among the different groups, whereas differences in continuous variables were evaluated using the Mann-Whitney U test. Survival analysis for the predefined primary endpoint was performed using the Kaplan-Meier method (18), and group comparisons were made using the log-rank test (19). Bar charts were created for each year to compare the rates of a composite endpoint, including local cancer recurrence, newly diagnosed metastases, and death between the low and high EtCO_2_ groups throughout the respective follow-up periods. To compare major differences in mean EtCO_2_ values, the entire patient cohort was sorted by ascending mean intraoperative EtCO_2_ values and then stratified into five groups for further survival analysis using the Kaplan-Meier method. Furthermore, a subgroup analysis was conducted to account for the duration of the respective EtCO_2_ values. Therefore, patients were categorized into three subgroups according to their duration of surgery. The high and low EtCO_2_ groups within these subgroups were subsequently re-evaluated for recurrence-free survival using Kaplan-Meier method. Thereafter, the primary outcome was analyzed by the Cox proportional hazard model (20), in which the effect of mean EtCO_2_ on recurrence-free survival was estimated after adjusting for the following covariates: age, gender, BMI, diabetes mellitus, smoking, UICC (tumor classification according to the Union for International Cancer Control) stage, epidural anesthesia, intraoperative sufentanil consumption and transfusions, resection margin status, tumor grade, and neoadjuvant, intraoperative, and adjuvant therapies. Using the Wald-Test, p-values for regression coefficients were obtained. Hazard ratios (HRs) were calculated using Cox analysis and presented with their respective 95% confidence intervals (CIs). Significance was defined as a two-sided p-value < 0.05. Survival rates at 1–5 years were assessed using the Kaplan-Meier technique, and statistical significance was evaluated employing the log-rank test. Prism 9.0.0 (GraphPad Prism Software, Inc., San Diego, CA) and IBM SPSS Statistics 28.0 (SPSS, Chicago, IL) and were used for statistical analysis and graphical representation.

## 3 Results

**Figure 1:**
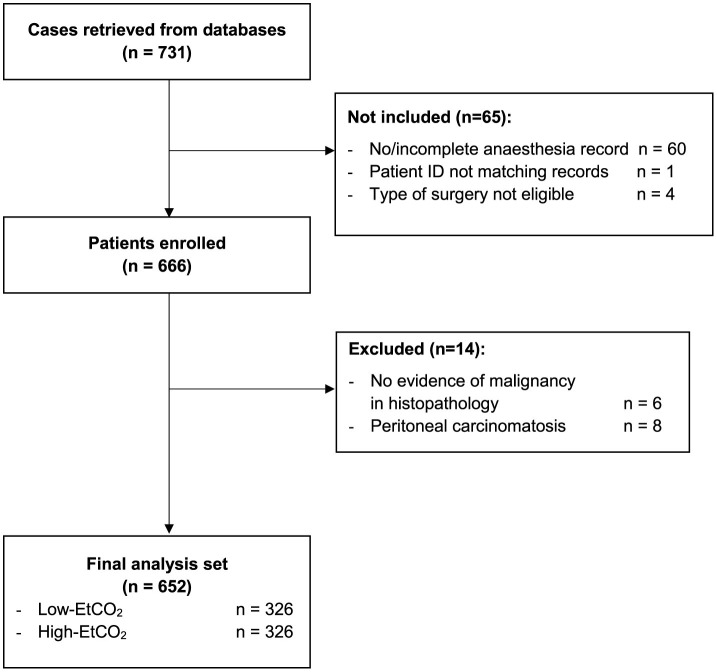
Participant flow chart. EtCO_2_, end-tidal carbon dioxide concentration; ID, identity document.

After applying the inclusion and exclusion criteria, the final analysis comprised 652 patients with pancreatic adenocarcinoma (Figure 1).

### 3.1 Patient characteristics

Table 1 and Supplementary Table 1 contain the primary clinical and demographic baseline characteristics. At the time of surgery, the mean age was 63 ± 10 years. The average BMI was 25.2 kg m^−2^ and 48.3% of patients were female.

**Table 1:** Clinical baseline characteristics of the study cohort.

**Variable**	**Analysis set**	**Low-EtCO_2_**	**High-EtCO_2_**	***p*-value**
	**(*****n*** = **652)**	**(*****n*** = **326)**	**(*****n*** = **326)**	
Age (years), mean ± SD	63.3 ± 9.9	63.1 ± 10.2	63.5 ± 9.6	0.784
Male, *n* (%)	337 (51.7)	172 (52.7)	165 (50.6)	0.583
BMI (kg/m^2^), mean ± SD	25.2 ± 3.8	25.2 ± 3.8	25.2 ± 3.7	0.974
**ASA status**, ***n*** **(%)**				
1–2	383 (58.7)	185 (56.7)	198 (60.7)	0.301
3–4	269 (41.2)	141 (43.3)	128 (39.3)	
**Smokers**, ***n*** **(%)**				
Active	135 (20.7)	67 (20.6)	68 (20.9)	0.902
Previous	55 (8.4)	26 (8.0)	29 (8.9)	
Diabetes mellitus, *n* (%)	150 (23.0)	75 (23.0)	75 (23.0)	>0.999
COPD, *n* (%)	35 (5.4)	20 (6.1)	15 (4.6)	0.385
Duration of surgery (min), median (Q1; Q3)	310 (245; 373)	310 (248; 375)	310 (245; 372)	0.949
Intensive care unit stay, *n* (%)	387 (59.4)	218 (66.9)	199 (61.0)	0.121
Duration of intensive care stay (d), mean ± SD	3 ± 7	6 ± 9	3 ± 4	0.813
Duration of hospitalization (d), mean ± SD	17 ± 12	17 ± 12	17 ± 11	0.339

Data are presented as mean ± SD or as absolute number (percentage). P-values refer to the comparison between low- EtCO_2_ vs. high- EtCO_2_ patients. Continuous data were compared using the Mann-Whitney U test. Categorical variables were compared using the chi-square test.

EtCO_2_, end-tidal carbon dioxide concentration; SD, Standard deviation; BMI, Body mass index; ASA, risk classification according to the American Society of Anesthesiologists; COPD, Chronic obstructive pulmonary disease.

There were no disparities between high and low EtCO_2_ levels in baseline characteristics or transfusion requirement. Furthermore, we noted no disparities concerning UICC stage, neoadjuvant therapy, resection margin and tumor grading status, or adjuvant therapy between groups. IORT was less prevalent in the high-EtCO_2_-group [8 (2.5%) vs. 21 (6.4%), high- vs. low-EtCO_2_, *p* = 0.014].

General anesthesia was performed as balanced anesthesia. In two participants (0.31%), fentanyl was administered as the intraoperative opioid, requiring the calculation of an equivalent dose to sufentanil (21). Epidural anesthesia was less prevalent in the high-EtCO_2_-group [230 (70.8%) vs. 258 (79.1%); high- vs. low-EtCO_2_, *p* = 0.014]. Intraoperative consumption of sufentanil was lower in the low-EtCO_2_-group (71.6 ± 34.7 vs. 76.7 ± 33.9; high vs. low-EtCO_2_, *p* = 0.014).

Surgery was always performed as open surgery in this observed study cohort.

### 3.2 Intraoperative EtCO_2_

After sorting the entire patient cohort in ascending order based on their mean intraoperative EtCO_2_ values, they were divided into two groups: the high- and low-EtCO_2_ groups, the determined cut-off value was 35.3 mmHg. The mean EtCO_2_ in the high-EtCO_2_ group was 36.8 mmHg ±1.9, compared to 33.8 mmHg ± 1.1 in the low-EtCO_2_ group.

After dividing the cohort into five groups, sorted according to the mean intraoperative EtCO_2_ value, mean EtCO_2_ values were 32.72 ± 0.97 mmHg, 34.26 ± 0.27 mmHg, 35.19 ± 0.26 mmHg, 36.09 ± 0.28 mmHg and 38.31 ± 2.45 mmHg, in ascending order.

### 3.3 Survival analysis

**Figure 2:**
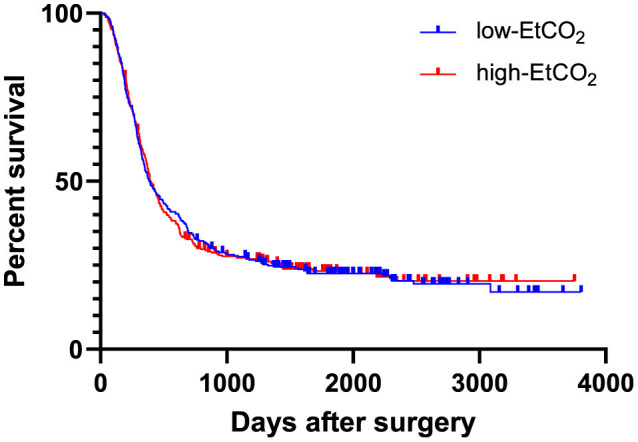
EtCO_2_ and recurrence-free survival. Patients were divided into low-EtCO_2_ group and high-EtCO_2_ groups. There was no difference in recurrence-free survival between the high and low-EtCO_2_ groups [HR 1.043 (95% CI: 0.875–1.243), log rank test: *p* = 0.909]. CI, confidence interval; EtCO_2_, end-tidal carbon dioxide concentration.

**Figure 3:**
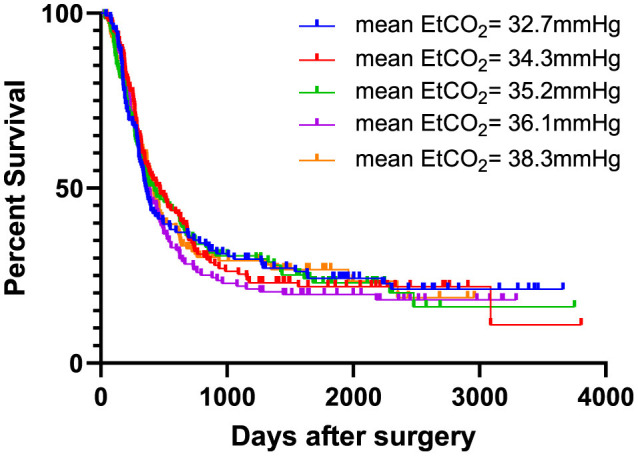
EtCO_2_ and recurrence-free survival stratified in five groups. Patients were categorized into five groups based on increasing mean EtCO_2_ values. There was no difference in recurrence-free survival between the five groups [HR 1.011 (95% CI 0.950–1.075), log rank test: *p* = 0.917]. EtCO2, end-tidal carbon dioxide concentration.

During the observation period, cancer recurrence was diagnosed in 493 patients (75.6%). Of these patients, 154 (31.2 %) had a local cancer recurrence, 298 (60.4 %) had distant metastases, and 32 patients (6.5 %) experienced both. Information regarding the nature of recurrence was not accessible for nine patients (1.8%). Throughout the observation period, 386 patients (59.2%) died. 373 patients (57.2%) succumbed to their cancer disease. Two patients (0.3%) passed away following a cardiovascular event. The reason for death was not available for 11 patients (1.7%). There was no difference in recurrence-free survival between the high and low-EtCO_2_ groups [HR = 1.043 (95% CI: 0.875–1.243), log rank test: *p* = 0.909] (Figure 2), nor did it differ among the groups when the patient cohort was divided into five groups [HR 1.011 (95% CI 0.950-1.075), log rank test: p = 0.917] (Figure 3). Bar charts revealed no differences in the occurrence of the composite endpoint, including local cancer recurrence, newly diagnosed metastases, and death between the high and low EtCO_2_ groups across the respective time periods (Supplementary Figure 1). Likewise, the subgroup analysis for different durations of surgery did not reveal differences between the respective high and low EtCO_2_ groups (Supplementary Figure 2).

Factors affecting the primary endpoint were studied via Cox analysis, which indicated no correlation between mean EtCO_2_ levels and recurrence–free survival [Coefficient −0.004, HR = 0.996 (95% CI: 0.95–1.04); *p* = 0.871] (Table 2). Variables that were associated with the primary endpoint included: BMI [Coefficient: −0.026, HR = 0.974 (95% CI: 0.950–0.999), p = 0.044], UICC stage IIb–III [Coefficient 0.402, HR = 0.669 (95% CI:0.523–0.855), p = 0.001], no grading due to adjuvant therapy [Coefficient: 0.421, HR = 0.656 (95% CI: 0.535–0.805), *p* < 0.001] and grading 3–4 [Coefficient 0.931; HR = 0.394 (95% CI: 0.201–0.774), *p* = 0.007].

**Table 2:** Independent effects of EtCO_2_ on recurrence-free survival.

	**Coefficient**	**SE**	***p*-value**	**HR**	**95% CI for HR**	
					**Lower**	**Upper**
Sex female	0.052	0.101	0.611	1.053	0.863	1.284
Age at time of surgery (y)	−0.006	0.005	0.251	0.994	0.985	1.004
BMI (kg/m^2^)	−0.026	0.013	**0.044**	0.974	0.950	0.999
No smoking (reference)			0.486			
Former smoking	0.135	0.127	0.286	1.145	0.893	1.467
Current smoking	0.015	0.198	0.940	1.015	0.688	1.497
Diabetes mellitus	0.092	0.115	0.421	1.097	0.876	1.373
Dose of sufentanil (μg)	0.003	0.002	0.114	1.003	0.999	1.007
Epidural anesthesia	−0.074	0.142	0.605	0.929	0.703	1.227
No RBC (reference)			0.253			
RBC (1–5 TU)	−0.859	0.607	0.157	0.423	0.129	1.391
RBC (6–10 TU)	−0.739	0.598	0.216	0.477	0.148	1.542
RBC (11–15 TU)	−0.396	0.605	0.512	0.673	0.206	2.202
RBC (>15 TU)	−1.196	0.773	0.122	0.302	0.067	1.375
PLT (1–5 TU)	−0.089	0.194	0.647	0.915	0.625	1.339
No FFP (reference)			0.683			
FFP (1–5 TU)	−0.770	1.171	0.511	0.463	0.047	4.596
FFP (>5 TU)	−0.931	1.146	0.417	0.394	0.042	3.727
UICC stage IIb-III	0.402	0.125	**0.001**	0.669	0.523	0.855
Grading G1-2 (reference)			**<0.001**			
No grading due to neoadjuvant therapy	0.421	0.104	**<0.001**	0.656	0.535	0.805
Grading G3-4	0.931	0.344	**0.007**	0.394	0.201	0.774
Resection margin status R1	0.244	0.125	0.050	1.277	1	1.630
Neoadjuvant chemotherapy	0.148	0.297	0.535	1.203	0.672	2.153
Neoadjuvant radiotherapy	0.084	0.356	0.813	1.088	0.541	2.188
IORT	0.623	0.341	0.068	1.864	0.955	3.638
Adjuvant therapy	−0.334	0.152	**0.027**	0.716	0.532	0.964
Mean EtCO_2_ (mmHg)	−0.004	0.023	0.871	0.996	0.953	1.042

The p-values of the regression coefficients were calculated using the Wald-Test. Hazard ratios estimated from the Cox analysis were reported as relative risks with corresponding 95% CIs. Boldface indicates p <0.05.

CI, Confidence interval; HR, Hazard ratio; SE, Standard error; BMI, Body mass index; UICC, Tumor classification according to the Union for International Cancer Control; IORT, intraoperative radiation therapy; EtCO_2_, End-tidal carbon dioxide concentration; RBC, Red blood cells; FFP, Fresh frozen plasma; PLT, Platelet concentrates; TU, Transfusion units.

### 3.4 Secondary endpoints

1-and 5-year survival rates did not differ between the high- and low-EtCO_2_ groups (1-year survival: 87.9% vs. 86.4%, high- vs. low-EtCO_2_, *p* = 0.855; 5-year survival: 31.1% vs. 25.2 %, high- vs. low-EtCO_2_, *p* = 0.855) (Table 3).

**Table 3:** Overall survival.

	**Analysis set**	**Low-EtCO_2_**	**High-EtCO_2_**	***p*-value**
	**(*****n*** = **652)**	**(*****n*** = **326)**	**(*****n*** = **326)**	
Number of patients alive after 1 year (1-year survival rate in %)	*n* = 639	*n* = 324	*n* = 315	0.833
	557 (87.2)	280 (86.4)	277 (87.9)	
Number of patients alive after 5 years (5-year survival rate in %)	*n* = 496	*n* = 270	*n* = 226	0.855
	125 (25.2)	84 (31.1)	41 (18.1)	

1-year and 5-year overall survival were estimated by the Kaplan Meier method. P-value refers to the log rank test.

EtCO_2_, End-tidal carbon dioxide concentration.

There were also no differences between the groups in terms of cardiovascular events, sepsis rate, surgical site infection rate or the rate of reoperations (Table 4).

**Table 4:** Secondary outcomes.

**Secondary outcome**	**Analysis set**	**Low-EtCO_2_**	**High-EtCO_2_**	**p-value**
	**(*****n*** = **652)**	**(*****n*** = **326)**	**(*****n*** = **326)**	
Cardiovascular event, *n* (%)	4 (0.6)	3 (0.9)	1 (0.3)	0.316
Reoperation during, *n* (%)	43 (6.6)	19 (5.8)	24 (7.4)	0.430
Sepsis, *n* (%)	3 (0.4)	2 (0.6)	1 (0.3)	0.563
SSI, *n* (%)	63 (9.7)	29 (8.9)	34 (10.4)	0.593
Superficial incisional	22 (3.4)	10 (3.1)	12 (3.7)	
Deep incisional	4 (0.6)	3 (0.9)	1 (0.3)	
Organ/space	37 (5.7)	16 (4.9)	21 (6.4)	

Categorial variables were compared using the chi-square test. Occurrence of myocardial infarction, cerebral infarction, or transitory ischemic attack during hospitalization were subsumed as cardiovascular events.

EtCO_2_, end-tidal carbon dioxide concentration; SSI, surgical site infection.

## 4 Discussion

In this retrospective cohort study, we did not identify a correlation between intraoperative EtCO_2_ and recurrence-free survival in patients undergoing pancreatic cancer surgery. This finding differs from our previous observation in other tumor entities (16), and it is in contrast our expectations that were based on *in vitro* studies (11, 12). BMI, UICC stage IIb-III, no grade due to adjuvant therapy, and grade G3-4 were independently associated with recurrence-free survival. In our secondary endpoint analysis, overall survival, incidence of cardiovascular events, sepsis, SSI, and need for reoperation did not differ between the high- and low-EtCO_2_ groups.

The correlation between EtCO_2_ levels and oncological outcome in pancreatic cancer patients has not yet been investigated. CO_2_ exerts diverse effects on tumor biology (2, 8–10). Effects of CO_2_ on mitochondrial metabolism (22), the cellular microenvironment (23), the expression of Vascular Endothelial Growth Factor (7), E-cadherin (7), and various matrix metalloproteinases (24, 25), which are known to influence cancer cell invasion and metastasis (26, 27), have been reported. Hypercapnic conditions in cervical cancer cells stimulate tumor cell proliferation. At the same time, hypercapnia led to a reduction in invasion, migration and adhesion (8, 9). However, potential effects of hypocapnic conditions remain uncertain. Exposing colon cancer cells to CO_2_ exhibits in heightened proliferation, adhesion disorder and elevated levels of growth factor expression (7, 10). The effect of CO_2_ on pancreatic cancer cells has rarely been studied. Nevler et al. exposed two pancreatic ductal adenocarcinoma cell lines to normocapnic (5% CO_2_) and hypercapnic (10% CO_2_) conditions (11). Hypercapnia led to increased tumor proliferation, radio resistance, and chemoresistance (11). In the first retrospective cohort study of 528 colorectal cancer patients investigating the association between intraoperative EtCO_2_ and oncological outcome, we demonstrated that lower EtCO_2_ levels were independently associated with enhanced recurrence-free survival (HR = 1.138, 95% CI:1.02-1.28, *p* = 0.027) at a median follow-up of 3.8 with an interquartile range of 2.5 to 5.1 years (16). The hazard of cancer recurrence decreased by 12.1% with each 1 mmHg reduce in mean EtCO_2_ (16). Surprisingly, in this study of patients who underwent pancreatic cancer surgery, EtCO_2_ level was not associated with recurrence-free survival. In addition to the various CO_2_-related effects on tumor biology in different tumor types (2, 8–10, 28), the biological effects of hypercapnia on tumor development are also discussed as a function of time and CO_2_ concentration (23). The EtCO_2_ values only differed by approximately 10% (3 mmHG) between the high and low EtCO_2_ groups. However, when patients were stratified into 5 groups, not even the extreme cases differed regarding recurrence-free survival.

Our study also found no differences in 1- and 5-year survival rates between the high and low EtCO_2_ groups. In contrast, in patients undergoing colorectal cancer surgery, both 1- and 5-year survival rates were higher in patients with lower EtCO_2_ values (16). The type of cancer itself significantly influences the average survival time of a patient (29–31). Individuals with pancreatic cancer typically experience a shorter average survival time compared to those with colorectal cancer (29–31). Factors such as tumor grading, UICC stage, or resection margin status appear to have a stronger influence on recurrence-free survival than EtCO_2_ in pancreatic cancer patients.

CO_2_ has been linked to multiple effects on the cardiovascular system. Hypocapnia can reduce the cerebral blood flow, impact the airway resistance, provoke cardiac arrythmias and trigger vasoconstrictions (15, 32, 33). Dony et al. demonstrated that hypocapnia, was linked to elevated 30-day postoperative mortality and extended hospitalization duration in patients undergoing non-cardiac surgery (32). In contrast, negative effects on the cardiovascular system due to hypercapnia have also been described. Increased blood CO_2_ levels can elevate the heart's oxygen consumption, potentially resulting in tachycardia and hypertension (34, 35). In this patient cohort, as well as in patients undergoing colorectal cancer surgery (16), intraoperative EtCO_2_ did not affect the incidence of cardiovascular events or the length of hospital stay.

Moreover, some authors suggest that hypercapnia has anti-inflammatory properties and improves tissue perfusion and oxygenation, potentially lowering the rate of SSI (34, 36–38). In our study, no association was observed between EtCO_2_ levels and SSI or the occurrence of sepsis. However, the incidence of sepsis and wound infections was low in this observed patient cohort.

Our study has several limitations that warrant consideration. The presence of digital anesthesia records constrained the duration of observation period, thereby limiting the sample size. Due to the study design, the representativeness, validity, and reliability of the results are limited. Moreover, the study design lacks the ability to exercise complete control over unmeasured biases.

The selection process for EtCO_2_ values lacked standardization, and the entire cohort was split into two equal groups based on the calculated mean EtCO_2_ values. The mean for the low EtCO_2_ group was in the mildly hypocapnic range, whereas the mean for the high EtCO_2_ group was in the normocapnic range. Despite comparing the intraoperative EtCO_2_ values by calculating individual mean values, we did not consider the individual dosages during surgery. The duration of EtCO_2_ levels was considered only in a subgroup analysis with different operation durations and not continuously. Additionally, inspiratory CO_2_ levels could not be considered. However, it is reasonable to assume that higher inspiratory CO_2_ levels would also result in elevated EtCO_2_ levels.

Because PaO_2_ values are determined irregularly in contrast to the continuous non-invasive measurement of EtCO_2_, we refrained from exploring the correlation between PaO_2_ and the measured EtCO_2_ values.

In conclusion, we demonstrated, that intraoperative EtCO_2_ during pancreatic cancer surgery is not associated with increased or decreased recurrence-free survival. EtCO_2_ was not associated with the secondary endpoints, namely overall survival, cardiovascular events, SSI, incidence of sepsis, or reoperations. During elective pancreatic cancer surgery, anesthesiologists should set EtCO_2_ targets for reasons other than oncological outcome until conclusive evidence from prospective, multicenter randomized controlled trials is available.

## Data Availability

The raw data supporting the conclusions of this article will be made available by the authors, without undue reservation.
