# Supplementary material for: Intraoperative end-tidal carbon dioxide levels are not associated with recurrence-free survival after elective pancreatic cancer surgery: a retrospective cohort study
Source: Front Med (Lausanne). 2024 Sep 11;11:1442283. doi: 10.3389/fmed.2024.1442283 (PMC11422119; doi:10.3389/fmed.2024.1442283)
Supplement: Supplementary file 1 [file Table_1.DOCX]

**Supplemental Table:** **Anaesthesiologic and tumour-specific characteristics of the study cohort**

| **Variable** | **Analysis Set**  **(n = 652)** | **Low-EtCO_2_**  **(n = 326)** | **High-EtCO_2_**  **(n = 326)** | **p-value** |
| --- | --- | --- | --- | --- |
| Epidural anaesthesia | 488 (75.0) | 258 (79.1) | 230 (70.8) | **.014** |
| Intraoperative dose of sufentanil (µg) | 74.1 ± 34.4 | 71.6 ± 34.7 | 76.7 ± 33.9 | **.014** |
| Intraoperative transfusion of RBC (TU) | 0.3 ± 1 | 0.4 ± 1.4 | 0.2 ± 0.9 | .135 |
| Intraoperative transfusion of FFP (TU) | 0.27 ± 1.1 | 0.33 ± 1.3 | 0.2 ± 0.86 | .337 |
| Intraoperative transfusion of PLT (TU) | 0.02 ± 0.17 | 0.01 ± 0.14 | 0.02 ± 0.19 | .991 |
| UICC stage, n (%)  UICC 0-2  UICC 3-4 | 194 (29.8)  457 (70.2) | 98 (30.1)  228 (69.9) | 96 (29.5)  229 (70.5) | .884 |
| Grading, n (%)  G1-2  G3-4  No grading due to neoadjuvant therapy | n=649  389 (59.9)  201 (31.0)  59 (9.1) | n=326  199 (61.0)  97 (29.8)  30 (9.2) | n=323  190 (58.8)  104 (32.2)  29 (9.0) | .796 |
| Resection margin status R1, n (%) | 476 (73.1) | 242 (74.2) | 234 (72.0) | .520 |
| Tumor localisation: Pancreatic head, n (%) | 504 (77.4) | 261 (80.1) | 243 (74.8) | .092 |
| Neoadjuvant chemotherapy, n (%) | 79 (12.1) | 38 (11.7) | 41 (12.6) | .708 |
| Neoadjuvant radiotherapy, n (%) | 29 (4.5) | 21 (6.4) | 8 (2.5) | .168 |
| IORT, n (%) | 29 (4.5) | 21 (6.4) | 8 (2.5) | **.014** |
| Adjuvant therapy, n (%) | n = 552  487 (88.2) | n = 274  235 (85.7) | n = 278  252 (90.6) | .075 |

Data are presented as mean ± SD, or as absolute number (percentage). P-values refer to comparison between low-EtCO_2_ vs. high- EtCO_2_ patients. Continuous data were compared using Mann-Whitney U test. Categorial variables were compared using chi-square test. Bold face indicates p-values<.05.

*EtCO_2_: end-tidal carbon dioxide concentration; SD: Standard deviation; UICC: Tumor classification according to the Union for International Cancer Control; IORT: Intraoperative radiation therapy; RBC: Red blood cells, FFP: Fresh frozen plasma, PLT: Platelet concentrates, TU: Transfusion units.*
