# Supplementary material for: Intraoperative end-tidal carbon dioxide levels are not associated with recurrence-free survival after elective pancreatic cancer surgery: a retrospective cohort study
Source: Front Med (Lausanne). 2024 Sep 11;11:1442283. doi: 10.3389/fmed.2024.1442283 (PMC11422119; doi:10.3389/fmed.2024.1442283)
Supplement: Supplementary file 2 [file Data_Sheet_1.docx]

**Supplemental Figure 1: Incidence of the composite endpoint (local cancer recurrence, newly diagnosed metastases, and death) over the years**


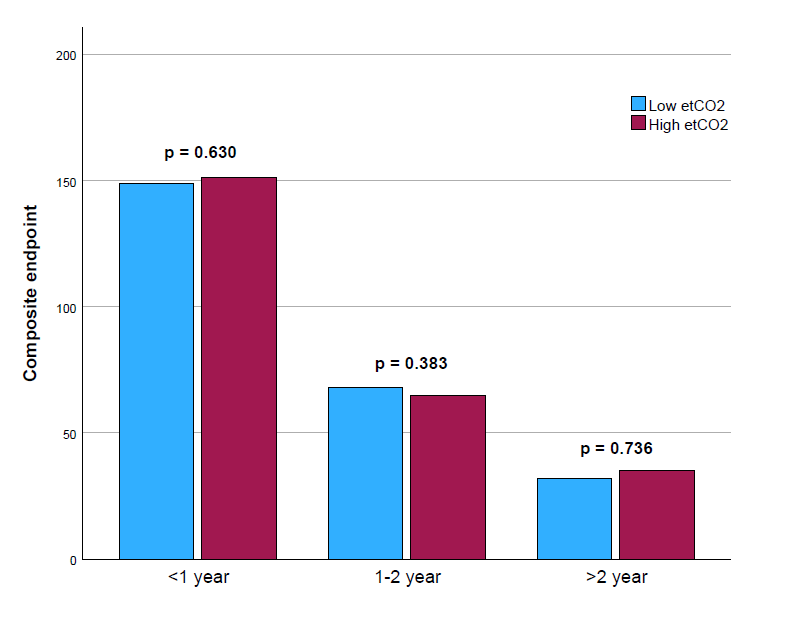

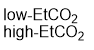


**Supplemental Figure 1: Incidence of the composite endpoint (local cancer recurrence, newly diagnosed metastases, or death) over the years**

Patients were divided into low-EtCO_2_ group and high-EtCO_2_ groups. There were no differences in the occurrence of local cancer recurrence, newly diagnosed metastases, or death between the high and low EtCO_2_ groups across the respective time periods.

*EtCO_2_: end-tidal carbon dioxide concentration.*
