# Supplementary material for: Intraoperative end-tidal carbon dioxide levels are not associated with recurrence-free survival after elective pancreatic cancer surgery: a retrospective cohort study
Source: Front Med (Lausanne). 2024 Sep 11;11:1442283. doi: 10.3389/fmed.2024.1442283 (PMC11422119; doi:10.3389/fmed.2024.1442283)
Supplement: Supplementary file 3 [file Data_Sheet_2.docx]

**Supplemental Figure 2:** **EtCO_2_ and recurrence-free survival for subgroups based on their duration of surgery**

**A)**


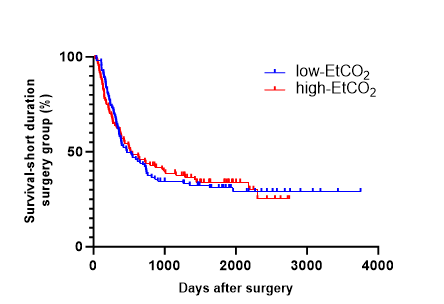


**B)**

**C)**

**Supplemental Figure 2: EtCO_2_ and recurrence-free survival for subgroups based on their duration of surgery**

A subgroup analysis was conducted to account for the duration of the respective EtCO_2_ values. Therefore, patients were categorized into three subgroups according to their duration of surgery (short, moderate, and long). The high and low EtCO_2_ groups within these subgroups were subsequently re-evaluated for recurrence-free survival using Kaplan-Meier method.

1. The median surgery time was 215 (Q1:166; Q3:245) min. The determined cut-off value for the high- and low-EtCO_2_ groups in this subgroup was 35.4 mmHg.
2. The median surgery time was 310 (Q1:290; Q3: 344) min. The determined cut-off value for the high- and low-EtCO_2_ groups in this subgroup was 35.4 mmHg.
3. The median surgery time was 406 (Q1:373; Q3: 452) min. The determined cut-off value for the high- and low-EtCO_2_ groups in this subgroup was 35.5 mmHg.

The subgroup analysis for different durations of surgery indicated no differences between the respective high and low EtCO_2_ groups (A: HR 0.98 (95% CI:0.897-1.070), log rank test: p = 0.940; B: HR 1.024 (95% CI:0.957-1.097), log rank test: p = 0.749; C: HR 0.996 (95% CI:0.740-1.341), log rank test: p = 0.980).

*EtCO_2_: end-tidal carbon dioxide concentration.*
